# Novel adulterants in unregulated opioids and their associations with adverse events

**DOI:** 10.17269/s41997-024-00990-7

**Published:** 2025-02-24

**Authors:** Samuel Tobias, Jennifer Angelucci, Evan Wood, Jane A. Buxton, Lianping Ti

**Affiliations:** 1 British Columbia Centre on Substance Use, Vancouver, BC Canada; 2 School of Population and Public Health, Faculty of Medicine, University of British Columbia, Vancouver, BC Canada; 3 Department of Medicine, Faculty of Medicine, University of British Columbia, Vancouver, BC Canada

**Keywords:** Adverse drug events, Benzodiazepines, Xylazine, Drug checking, Effets secondaires indésirables des médicaments, Benzodiazépines, Xylazine, Vérification des drogues

## Abstract

**Objective:**

In recent years, Canada’s unregulated drug supply has become permeated by novel adulterants (e.g., fentanyl analogues, benzodiazepines, xylazine). While fentanyl has been shown to be associated with overdose mortality and other non-fatal health outcomes, adverse events (AE) associated with these adulterants remain poorly described. This study seeks to identify whether common adulterants identified through drug checking services are associated with increased prevalence of specific adverse events reportedly experienced by people who use drugs.

**Methods:**

Drug checking samples were analyzed using Fourier-transform infrared spectroscopy and immunoassay strips at harm reduction sites in British Columbia. Self-reported AE (e.g., non-fatal overdose, prolonged sedation, seizures) were recorded from individuals who checked opioids post-consumption. Adjusted prevalence ratios (aPR) and 95% confidence intervals (95% CI) of AE among common adulterants were calculated using generalized linear models with a Poisson distribution, controlled for presence of other adulterants, expected drug, geographic location, and month.

**Results:**

Between February 2022 and May 2024, 80,415 samples were analyzed at community sites. Among eligible samples, 36.1% were expected opioids, 42.2% of which were checked post-consumption. AE were noted among 10.7% of post-consumption opioid drug checks. After adjustment, the presence of benzodiazepines in opioid samples was associated with increased prevalence of any AE (aPR 1.97; 95% CI 1.70–2.27), as was the presence of xylazine (aPR 1.50; 95% CI 1.09–2.07). Considering specific AE, benzodiazepines were associated with increased prevalence of overdose (aPR 2.05; 95% CI 1.68–2.51) and prolonged sedation (aPR 3.35; 95% CI 2.54–4.43).

**Conclusion:**

Non-fatal AE associated with unregulated opioids have been largely undescribed. Our findings report specific AE associated with different adulterants in the unregulated opioid supply. With this information, tailored public health interventions and services focused on these adulterants can be developed.

## Résumé

**Objectif:**

Ces dernières années, de nouveaux adultérants (analogues du fentanyl, benzodiazépines, xylazine) se sont infiltrés dans l’approvisionnement non réglementé en drogues du Canada. Le fentanyl est associé à des mortalités par surdose et à d’autres résultats cliniques non mortels, mais les effets indésirables (EI) associés à ces adultérants sont encore mal décrits. Nous avons voulu déterminer si les adultérants couramment identifiés par les services de vérification des drogues sont associés à la prévalence accrue d’effets indésirables particuliers subis par les personnes faisant usage de drogues.

**Méthode:**

Nous avons analysé des échantillons de vérification des drogues par spectroscopie infrarouge à transformée de Fourier et par bandes d’immunoessai dans des sites de réduction des méfaits de la Colombie-Britannique. Nous avons enregistré les EI (surdoses non mortelles, sédation prolongée, convulsions) déclarés par les personnes ayant vérifié les opioïdes après consommation. Nous avons calculé les ratios de prévalence ajustés (RPa) et les intervalles de confiance de 95% (IC de 95%) des EI associés aux adultérants courants à l’aide de modèles linéaires généralisés avec une distribution de Poisson, en tenant compte de la présence d’autres adultérants, de la drogue ou du médicament attendu, du lieu géographique et du mois.

**Résultats:**

Entre février 2022 et mai 2024, nous avons analysé 80,415 échantillons dans des sites communautaires. De tous les échantillons admissibles, 36,1% étaient des opioïdes attendus, dont 42,2% avaient été vérifiés après consommation. Des EI ont été observés dans 10,7% des vérifications d’opioïdes après consommation. Après l’apport d’ajustements, la présence de benzodiazépines dans les échantillons d’opioïdes était associée à la prévalence accrue d’un EI (RPa 1,97; IC de 95% 1,70–2,27), tout comme la présence de xylazine (RPa 1,50; IC de 95% 1,09–2,07). Pour les EI particuliers, les benzodiazépines étaient associées à la prévalence accrue de surdoses (RPa 2,05; IC de 95% 1,68–2,51) et à la sédation prolongée (RPa 3,35; IC de 95% 2,54–4,43).

**Conclusion:**

Les EI non mortels associés aux opioïdes non réglementés ne sont pas décrits pour la plupart. Nos constats font état d’EI particuliers associés à différents adultérants dans l’approvisionnement non réglementé en opioïdes. Ces informations permettront de mettre au point des interventions et des services de santé publique adaptés à ces adultérants.

## Introduction

Canada continues to contend with a drug poisoning crisis driven by increasingly adulterated illicit drug markets. Unregulated opioids (that is, opioid mixtures prepared for sale on the unregulated, illicit market) are primarily composed of fentanyl and its analogues (Ciccarone, 2017; Crepeault et al., 2023; Mars et al., 2019), and, depending on the specific setting, adulterated with other substances such as benzodiazepines or xylazine, a veterinary anesthetic (Friedman et al., 2022; Laing et al., 2021; Zhu, 2023). In British Columbia (BC), the province with the highest mortality rates from drug toxicity in Canada (Public Health Agency of Canada, 2023), 85% of individuals who died from unintentional drug toxicity in 2023 were found to have fentanyl in their system (BC Coroners Service, 2024). In addition to fentanyl, novel adulterants found in BC’s unregulated opioids likely pose unique risks and harms to people who consume them, either intentionally or unintentionally. Of increasing concern in BC is the adulteration of unregulated opioids with benzodiazepines, which were found in 42.7% of post-mortem toxicology results among BC unregulated drug deaths in 2023 (BC Coroners Service, 2024). Health Canada’s Drug Analysis Service, which analyzes seized samples for BC law enforcement agencies, reported a 40% increase in province-wide detection of benzodiazepines in 2023 over 2022 (Government of Canada, 2024), compounding existing upward trends. While not as prevalent in BC, xylazine-related harms are well documented in the United States (Friedman et al., 2022). Both benzodiazepines and xylazine are depressants that, when combined with opioids, can synergistically increase the risk of drug toxicity (Friedman et al., 2022; Laing et al., 2021; Russell et al., 2023). Fluorofentanyl, a fluorinated analogue of fentanyl with slightly reduced potency (Truver et al., 2022), became common in BC’s unregulated opioids in 2022 (Knill et al., 2023). However, its specific effects and any distinct health outcomes compared to fentanyl remain largely unknown. In the context of an ongoing drug poisoning crisis, where a small fraction of drug use results in death, it remains important to identify elements of the unregulated drug supply associated with non-fatal adverse outcomes which contribute to drug-related morbidity, and may be risk factors for future mortality (Caudarella et al., 2016).

An adverse event (adverse drug reaction) consists of a harmful, unpleasant, or unexpected reaction resulting from the use of a medicinal product (Coleman & Pontefract, 2016). Identifying adverse events associated with newly approved medications is important for patient safety and comprises tracking side effects and adverse events (referred to as phase four of a clinical trial) (Suvarna, 2010). While these events are reported to and logged in national and international databases as a part of ongoing regulatory practices for medications, people who use unregulated drugs rely on word of mouth within their communities or networks to share drug safety information (Latkin et al., 2004; Valente et al., 2019). In the context of a rapidly evolving unregulated drug supply where the contents of drugs change and are distributed with unknown concentrations, little is known about adverse events associated with unregulated drugs outside of controlled medical settings.

Drug checking services offer a unique ability to engage directly with people who use drugs and ask them questions about their drug use. A harm reduction service at their core, drug checking services have become an important public health intervention to inform individuals about the contents of their drugs so they can make informed decisions about how they wish to use, sell, or share their drugs. While intended to be performed prior to consuming the drug, post-use drug checking can help individuals identify why an adverse event associated with a particular sample may have occurred. The interaction of a drug check therefore offers a way to relate specific drug samples to peoples’ subjective experience of using a sample. Indeed, previous research in BC has indicated that 30.8% of stimulants and 39.2% of opioids were checked after the service user had already consumed some of that particular drug (Beaulieu et al., 2021). Identifying associations between drug sample contents and adverse events experienced by people who use drugs may offer public health decisionmakers crucial information that can shape treatment provision and substance use programs or policies. Using the available point-of-care drug checking data from BC, this study seeks to identify whether common adulterants identified through drug checking services are associated with increased prevalence of specific adverse events reportedly experienced by people who use drugs.

## Methods

Drug checking services have been offered at BC harm reduction sites since October 2017. Service users can anonymously submit a small sample of their drugs to be analyzed with Fourier-transform infrared (FTIR) spectroscopy plus fentanyl and benzodiazepine immunoassay strips. Results and tailored harm reduction advice are returned to the service user in as little as 5 min. Results of the drug check, including what the expected drug was and whether the drugs were being checked pre- or post-consumption (e.g., “Have you used any of this drug yet?”), were logged in an electronic data capture system by trained drug checking technicians. The electronic data capture system additionally collects location and timing information for each drug check. In February 2022, as a part of public health monitoring effort, all drug checking technicians were instructed to ask whether a sample being checked post-consumption was associated with any adverse events. The list of optional adverse events was determined from surveying harm reduction organizations across the province and was approved by public health partners in BC. Ethics approval was obtained through the Providence Health Care/University of British Columbia Research Ethics Board as part of a larger drug checking program evaluation.

### Statistical analysis

We first calculated descriptive statistics for the drug checking samples that met criteria for inclusion. To be included, samples must have been analyzed at point-of-care drug checking sites in BC between February 2022 and May 2024. We excluded samples that were submitted by mail to ensure the service user was asked about history of adverse events. Samples could have more than one adverse event reported. The remaining analyses were conducted on samples expected to be opioids by the service user (e.g., fentanyl, heroin, “down”, oxycodone) that were checked post-consumption. We determined the presence of adulterants of interest (i.e., fentanyl, fluorofentanyl, benzodiazepines including etizolam, and xylazine) in samples if the compound was detected by FTIR spectroscopy or by the use of an immunoassay strip in the case of fentanyl and benzodiazepines.

### Prevalence ratios

Unadjusted and adjusted prevalence ratios (PR; aPR) with 95% confidence intervals (95% CI) for any and specific adverse events were calculated among the common adulterants using generalized linear models with a Poisson distribution and log link. For the multivariable models, we adopted a forward variable selection approach based on Akaike’s information criterion (Akaike, 2011), comparing each fuller model to its reduced counterpart. This approach resulted in aPR controlled for calendar month, city or town where the drug check took place, and the expected drug reported by the service user. All analyses were conducted in R (version 4.2.1) (R Core Team, 2001) using RStudio (version 2024.04.1 + 748) (RStudio Team, 2023).

## Results

**Fig. 1:**
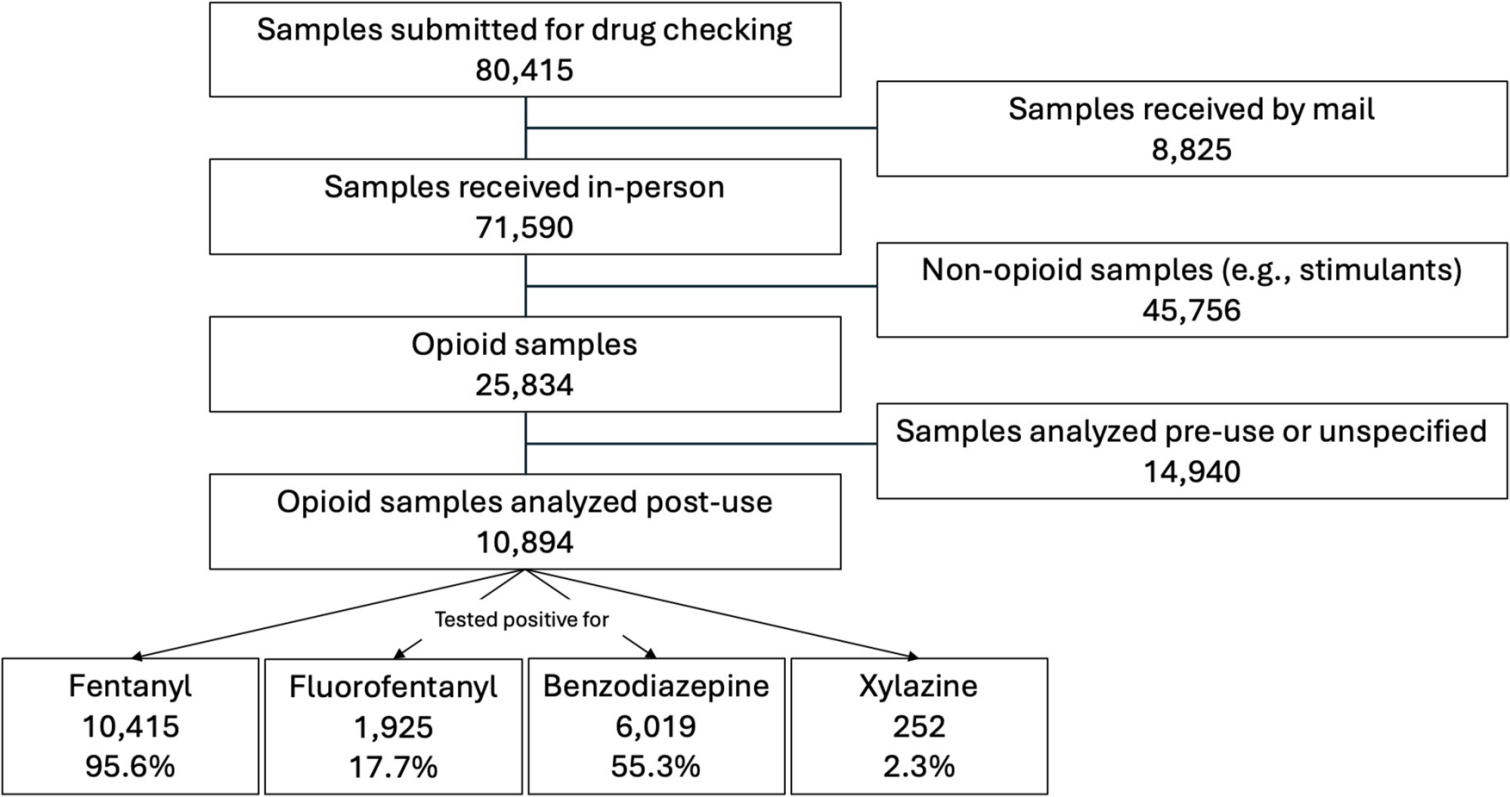
Flowchart depicting eligible drug checking samples submitted to British Columbia community harm reduction sites

Over the course of the study period, 80,415 drug samples were analyzed at drug checking sites in BC using FTIR spectroscopy and immunoassay strips. Among the samples received in-person, 25,834 (36.1%) were expected to be opioids by the service user, and 10,894 (42.2%) of these opioids were submitted for drug checking post-consumption (Fig. 1). Among the post-use opioid samples, 1167 (10.7%) were reportedly associated with at least one adverse event (Table 1). Of these opioid samples, 10,415 (95.6%) tested positive for fentanyl, 6019 (55.3%) tested positive for a benzodiazepine, 1925 (17.7%) tested positive for fluorofentanyl, and 252 (2.3%) tested positive for xylazine (Fig. 1).

**Table 1:** Counts of self-reported adverse events by users of drug checking services in British Columbia, Canada, among 10,894 opioid samples analyzed after consumption by the service user, February 2022 through May 2024

Adverse event	Count
Fatal opioid toxicity (overdose)	652
Non-fatal opioid toxicity (overdose)	612
Blackout (including memory loss)	235
Drowsiness or sedation	97
Dizziness, nausea, vomiting, headaches, blurred vision	78
Catatonia (inability to move)	60
Confusion	50
Seizure	22
Hallucinations (auditory or visual)	21
Allergic reaction, itchiness, numbness	20
Agitation (medium, including uncontrolled movements)	16
Agitation (minor, including anxiety)	13
Chest tightness	11
Agitation (major, including violent movements)	10
Strong or unexpected effects	20
Irregular heartbeat	6
Other (please specify)^a^	31

^a^Reported “other” adverse events that were consistent with existing categories were recoded into the respective category

More than one adverse event could be reported per sample

After adjustment, the presence of benzodiazepines was associated with a 97% increased prevalence of a reported adverse event (aPR 1.97; 95% CI 1.70–2.27), and xylazine a 50% increased prevalence of a reported adverse event (aPR 1.50; 95% CI 1.09–2.07). Neither fentanyl nor fluorofentanyl was associated with a change in adverse event reporting prevalence (Table 2). When we examined individual adulterants and specific adverse events (Table 2), benzodiazepines were associated with a 105% increased prevalence of an overdose-related adverse event (aPR 2.05; 95% CI 1.68–2.51), a 120% increased prevalence of dizziness or confusion (aPR 2.20; 95% CI 1.41–3.41), a 234% increased prevalence of agitation (aPR 3.34; 95% CI 1.49–7.46), and a 235% increase in the prevalence of prolonged sedation (aPR 3.35; 95% CI 2.54–4.43). Xylazine presence was associated with a 126% increase in prevalence of prolonged sedation (aPR 2.26; 95% CI 1.39–3.67) and a 627% increased prevalence of seizure events (aPR 7.27; 95% CI 1.56–33.34). In contrast, fentanyl presence was associated with a decreased prevalence of reported agitation-related events (aPR 0.11; 95% CI 0.03–0.36).

**Table 2:** Unadjusted and adjusted prevalence ratios for self-reported, specific adverse events calculated using logistic regression (Poisson distribution with log link). Opioid samples (*n* = 10,894) were received for drug checking at harm reduction sites in British Columbia, Canada, between February 2022 and May 2024 and were reported to have been already consumed by the service user

Adulterant	Prevalence ratio (95% CI)	*p*-value	Adjusted prevalence ratio^a^ (95% CI)	*p*-value
Any adverse event				
Benzodiazepine	2.20 (1.93–2.50)	< 0.01*	1.97 (1.70–2.27)	< 0.01*
Fentanyl	1.10 (0.82–1.47)	0.54	0.87 (0.58–1.32)	0.52
Fluorofentanyl	0.77 (0.65–0.90)	< 0.01*	0.90 (0.76–1.07)	0.23
Xylazine	1.62 (1.19–2.19)	< 0.01*	1.50 (1.09–2.07)	0.01*
Overdose (fatal and non-fatal combined)				
Benzodiazepine	2.80 (2.33–3.36)	< 0.01*	2.05 (1.68–2.51)	< 0.01*
Fentanyl	3.28 (1.70–6.32)	< 0.01*	1.39 (0.64–3.01)	0.40
Fluorofentanyl	0.83 (0.67–1.03)	0.08	0.91 (0.73–1.14)	0.43
Xylazine	1.27 (0.81–2.01)	0.30	1.02 (0.63–1.67)	0.93
Prolonged sedation (drowsiness, blackout, catatonia combined)				
Benzodiazepine	3.43 (2.64–4.46)	< 0.01*	3.35 (2.54–4.43)	< 0.01*
Fentanyl	4.10 (1.53–10.99)	< 0.01*	1.48 (0.53–4.13)	0.46
Fluorofentanyl	0.80 (0.60–1.07)	0.14	1.00 (0.73–1.35)	0.98
Xylazine	2.22 (1.38–3.56)	< 0.01*	2.26 (1.39–3.67)	< 0.01*
Dizziness and confusion				
Benzodiazepine	1.28 (0.90–1.84)	0.18	2.20 (1.41–3.41)	< 0.01*
Fentanyl	0.24 (0.15–0.38)	< 0.01*	0.42 (0.17–1.02)	0.05
Fluorofentanyl	0.50 (0.28–0.90)	0.02*	0.67 (0.36–1.23)	0.20
Xylazine	1.05 (0.34–3.27)	0.94	1.29 (0.41–4.07)	0.66
Agitation (minor, medium, major)				
Benzodiazepine	1.69 (0.85–3.35)	0.14	3.34 (1.49–7.46)	< 0.01*
Fentanyl	0.24 (0.10–0.57)	< 0.01*	0.11 (0.03–0.36)	< 0.01*
Fluorofentanyl	0.90 (0.38–2.16)	0.82	1.21 (0.49–2.96)	0.68
Xylazine	1.17 (0.16–8.52)	0.87	1.49 (0.20–11.01)	0.70
Seizure^b^				
Benzodiazepine	1.42 (0.60–3.38)	0.43	1.05 (0.40–2.74)	0.98
Fluorofentanyl	1.04 (0.35–3.06)	0.95	1.23 (0.40–3.76)	0.74
Xylazine	4.22 (0.99–17.97)	0.05	7.27 (1.56–33.34)	0.01*

^*^ indicates significance at the *p* < 0.05 level

^a^Adjusted prevalence ratio controlled for other adulterants present, expected drug, city or town where drug check took place, and calendar month

^b^Fentanyl was excluded from this model due to a zero cell count in fentanyl-negative, seizure-related samples

## Discussion

We found an increased prevalence of reported adverse events following the consumption of unregulated opioids that tested positive for benzodiazepines or xylazine. Outside of clinical settings, the association between the adulteration of opioids with benzodiazepines and the effects of prolonged sedation has been largely assumed. Our findings offer important evidence towards this relationship, key to preventing the negative outcomes associated with prolonged sedation. Prolonged sedation from unintentional benzodiazepine or xylazine exposure due to adulterated opioids is a medical emergency (BCCDC Harm Reduction Services, 2023). It carries a risk of hypoxic brain injury and can make it difficult for responders to distinguish between prolonged sedation and severe opioid toxicity. It further leaves individuals vulnerable to theft or violence as they are rendered unresponsive and may have inability to react (Russell et al., 2023). Understanding the association between drug adulteration and the potential for adverse events is essential for improving outcomes by developing effective harm reduction interventions (e.g., the expansion of peer-led oximeter use during prolonged sedation (Moe et al., 2024)) and tailored drug checking result reporting. Our findings not only have potential to inform public health interventions but could also serve as an advisory tool for people who use drugs and frontline community health workers, highlighting the risks associated with specific adulterants in pre-consumption settings. While further research is needed to determine causal pathways and refine this advice, noting the heightened risks associated with drugs such as benzodiazepines or xylazine can aid in community health promotion and proactive harm reduction efforts, especially in settings where word of mouth often serves as a primary information channel (Latkin et al., 2004).

Of additional note, xylazine was associated with increased prevalence of reported seizures. Although among only a small sample size, this significant association highlights an important area for directed observation to see whether this manifests in clinical settings. Xylazine has been linked to skin ulcers and soft tissue infections, particularly in settings with high prevalence in the unregulated drug supply, such as the northeastern United States (Friedman et al., 2022; Malayala et al., 2022). While not an acute adverse event akin to the others reported in this study, further observation of this chronic effect of xylazine exposure is warranted in BC. Finally, fentanyl has been the predominant opioid in the BC unregulated opioid supply since at least 2017 (Crepeault et al., 2023), which may explain the lack of positive associations between fentanyl or fluorofentanyl and reported adverse events when controlled for other adulterants. As people who use unregulated opioids may be expecting its effects, fentanyl’s decreased association with the reporting of agitation-related adverse events likely relates to its sedating qualities and speaks to its being the principal drug of choice among people who use unregulated opioids in BC (Karamouzian et al., 2020).

Outside of a clinical trial setting, adverse events are typically reported spontaneously and logged in large databases (Coleman & Pontefract, 2016; Rothman et al., 2004). Due to the nature of spontaneous reporting to regulatory authorities, it is not possible to determine how many times medications are consumed without an incident adverse event. This absence of a denominator necessitates the calculation of specific statistics such as the reporting odds ratio or a proportional reporting ratio, which are used as signal detectors of association, initiating an investigation if a threshold is reached (EudraVigilance Expert Working Group, 2006). In our study, all post-consumption users of drug checking services were asked whether their sample had been associated with an adverse event. This therefore captured individuals who responded that the sample had not caused an adverse event, providing us an adequate denominator to directly calculate self-reported aPR, and representing a significant strength of our study.

Our study has limitations that must be considered. First, drug checking in BC is anonymous and therefore it is not possible to determine patterns in individuals’ drug checking behaviours, such as frequency or repeat checking. Second, reporting of drug check timing (pre- or post-consumption) and the reporting of an adverse event may be subject to information biases or measurement error. For instance, some people who use opioids may purposefully pursue prolonged sedation as a drug effect, which might otherwise be considered a serious adverse event to a health care provider. Of note, the most common adverse event reported in our study was fatal overdose. This may be due to service users’ misinterpretation of the question posed by the trained drug checking technician (confusing the specific sample with the broader “batch” or other drugs of similar appearance), but is at least partly explained by third-party drug checking (drug checking on someone else’s behalf), which is common in BC (Larnder et al., 2021). This has the potential to lead to over-reporting of adverse events; however, those who experienced a fatal adverse event are not directly included in our study. Similarly, an individual’s experiences of adverse events may drive that person to use drug checking, overrepresenting remarkable or unusual samples. Third, due to limitations in the sensitivity and specificity of drug checking technologies (McCrae et al., 2020), there exists the possibility of misclassification of adulterant presence. This is likely most relevant to xylazine presence, as the FTIR may miss xylazine if present at low concentrations (Tobias et al., 2020). Other high-potency opioids (e.g., carfentanil, nitazenes) may also have gone undetected by the drug checking technologies used but have still contributed to adverse event occurrences. As every community and local drug supply differs, these findings may not be generalizable to other settings.

## Conclusion

The prevalence of specific adverse events associated with adulterants in the unregulated drug supply has been largely underreported. This study begins to provide evidence towards relationships between specific adulterants and the occurrence of adverse events, which have generally been anecdotal outside of controlled clinical settings. Identifying the association between specific adverse events and the presence of adulterants in the unregulated drug supply is important for advancing evidence-based harm reduction strategies and may help inform health care policy in the context of this ongoing crisis.

## Contributions to knowledge

What does this study add to existing knowledge?

- This study contributes new insights into the drug poisoning crisis by documenting the prevalence of reported adverse events associated with specific adulterants in the unregulated drug supply, a topic that has been underexplored in previous research.
- By identifying and analyzing the relationship between adverse events and specific adulterants, this study moves beyond anecdotal evidence to provide empirical support for understanding how drug adulteration impacts public health.

What are the key implications for public health interventions, practice, or policy?

- Understanding the association between drug adulteration and the potential for adverse events is essential for improving outcomes by developing effective harm reduction interventions and tailored drug checking result reporting.
- These research findings can facilitate early recognition and response to adverse events associated with adulterated drugs.

## Data Availability

Data underlying this study are not available for distribution.

## Code availability

Code underlying this study is not available.
